# Using quantitative risk information in decisions about statins: a qualitative study in a community setting

**DOI:** 10.3399/bjgp15X684433

**Published:** 2015-03-30

**Authors:** Louisa Polak, Judith Green

**Affiliations:** Department of Health Services Research and Policy, London School of Hygiene and Tropical Medicine, London, and North Hill Medical Group, Colchester.; Department of Health Services Research and Policy, London School of Hygiene and Tropical Medicine, London.

**Keywords:** decision making, shared, general practice, prevention, risk, statins, HMG-CoA

## Abstract

**Background:**

A large literature informs guidance for GPs about communicating quantitative risk information so as to facilitate shared decision making. However, relatively little has been written about how patients utilise such information in practice.

**Aim:**

To understand the role of quantitative risk information in patients’ accounts of decisions about taking statins.

**Design and setting:**

This was a qualitative study, with participants recruited and interviewed in community settings.

**Method:**

Semi-structured interviews were conducted with 34 participants aged >50 years, all of whom had been offered statins. Data were analysed thematically, using elements of the constant comparative method.

**Results:**

Interviewees drew frequently on numerical test results to explain their decisions about preventive medication. In contrast, they seldom mentioned quantitative risk information, and never offered it as a rationale for action. Test results were spoken of as objects of concern despite an often-explicit absence of understanding, so lack of understanding seems unlikely to explain the non-use of risk estimates. Preventive medication was seen as ‘necessary’ either to treat test results, or because of personalised, unequivocal advice from a doctor.

**Conclusion:**

This study’s findings call into question the assumption that people will heed and use numerical risk information once they understand it; these data highlight the need to consider the ways in which different kinds of knowledge are used in practice in everyday contexts. There was little evidence from this study that understanding probabilistic risk information was a necessary or valued condition for making decisions about statin use.

## INTRODUCTION

Statin prescriptions for those at high cardiovascular risk have widely been seen as an important contributor to reducing the population burden of heart disease.^1^ Such strategies rely on GPs identifying those at higher risk, and communicating the benefits of medication to patients. A large body of research now addresses how to communicate risk information,^2^ and a growing evidence base is emerging on how best to present patients with quantitative information about risk in general practice.^3^–^6^ This evidence suggests that information about risks is best presented quantitatively, as event rates,^2^ although GPs may prefer qualitative rather than quantitative presentations of risk.^3^ Evidence that presenting numeric information to patients improves risk comprehension^4^ has influenced good-practice guidance for developing decision aids for patients.^6^

Most evaluative research about risk communication formats has used accurate recall of information and comprehension as end points.^2^,^4^,^7^ However, there is less research about how such cognitive risk understanding is used in practice; that is, once people have the information and comprehend it, how will they use this knowledge to make decisions? Patients’ general views of preventive medication use have been widely researched, with reported reservations about taking hypertensive medication^8^ reflecting reservations found for long-term and preventive medications in general.^9^ In the light of recent interest in risk communication, there is a need for more research on the specific question of what use is made of quantitative risk information in decision making about preventive medication. This article addresses this question through analysis of qualitative data generated in a study of how people make decisions about taking statins.

## METHOD

### Setting and participants

Thirty-four participants aged >50 years were recruited and interviewed face-to-face in community settings, most in their homes, in East Anglia between 2011 and 2013. Invitations to participate were made through community groups such as lunch clubs and an exercise class, and snowballing from initial participants to identify those offered statins. All participants had been offered a statin for either primary (*n* = 17) or secondary prevention (*n* = 17); over half (*n* = 22) were currently taking statins. Participants were aged 53–87 years.

### Data generation

Twenty-two participants were interviewed in couples, and 12 in individual interviews. An advantage of pair interviews, which generate less formal accounts than are often generated in one-to-one interviews, is that they provide some access to how participants talk within the everyday contexts within which decisions are made. Paired interviews also have an advantage over private interviews as they allow participants to discuss issues that may be sensitive within relationships (such as fear of future ill health). All interviews were conducted by the one author, who introduced herself as a local GP doing a research project that was separate from her practice work. None of the interviewing GP’s patients were invited to participate. All interviews were recorded and transcribed verbatim. Interviews were semi-structured, using a brief topic guide, and lasted 23–87 minutes. Prompts included questions on participants’ state of health, where their knowledge about health came from, how they looked after their health, use of medication, and deciding whether to take statins.

**How this fits in**

Much is written about the best way to present quantitative risk information to patients, as clinicians are encouraged to do. Less research considers how such information is utilised in practice. In this study of interviewees’ accounts of their decisions about statins, risk estimates were hardly mentioned and never described as influential. If replicated elsewhere, this raises the question whether communicating quantitative risk information should remain a central component of endeavours to facilitate shared decision making.

### Analysis

Analysis drew on elements of the constant comparative method,^10^ beginning with in-depth line-by-line coding of early data, and comparisons within the data and with the literature to generate provisional explanations. Coding and memos were recorded using multiple Microsoft^®^ Word documents. Codes were refined by relating them to subsequent batches of data and further reading of relevant literature, in an iterative process aiming to build a robust and potentially generalisable account: ‘a plausible story about what is going on’.^11^ Throughout this process, rigour was increased by regular discussion about coding decisions and analytic direction. In this study, all names are pseudonyms and identifying material has been removed.

## RESULTS

Initial thematic analysis elicited a surprising fact: although people’s accounts were full of numbers describing health or health behaviours, there were almost no instances where numbers were used to talk about risk or prevention. Further analysis was directed towards explaining this contrast, by comparing the way numerical test results were used with the way risk and prevention were talked about.

### Using quantitative information: ‘magic numbers’

Participants’ accounts contained varied and frequent references to numbers related to health and health care, including numeric indicators of weight, blood pressure, medication dosage, and blood test results. A common use of numbers was for making comparative assessments in relation to, for instance, their past self, or other people:

*‘All I know is my blood pressure is a lot lower than it used to be, because it used to be 180/90 … and is now 118/60.*’

(Peter)

A second use of numeric values was as triggers for action. Here, participants used specific numbers as thresholds or goals when talking about the way they took decisions about health behaviours or healthcare use:

*‘Since I’ve been on the statins it’s down to about 2 or 2.5, which is where they want it to be with my situation.*’

(Larry)

*‘They said it was about 6.9 and they put me on Bezalip^®^.*’

(Debbie)

These examples are typical in that the rationale for choosing a particular number as a goal or trigger was seldom mentioned or queried. Often the source for the figure was (as in these examples) a generic ‘they’ rather than a specific doctor, and the value itself was recalled hesitantly, suggesting uncertainty:

Claire: *They put* [Walter] *on statins a few months ago and I think his cholesterol is what, 2.9?*’

Walter: *‘4.2 … that is good or something, I don’t know what it all means, but 4.*’

However, if the rationale for numbers was mysterious, they were tacitly accepted, even powerful, forces for eliciting action. As Henry’s mention of the ‘magic’ power of ‘5’ suggests:

*‘Every time it has been mentioned it has been below the magic figure, the 5 ... 5.5, I can’t remember which it is, anyway whatever it is there is a threshold and it has been below the threshold.*’

(Henry)

That such numbers had an esoteric element did not mean patients were resistant to scientific evidence. Indeed, the roots of the numbers in ‘hard evidence’ were an essential factor in their authority for some, as suggested by Don’s citation of ‘chemistry’ in his exchange with his wife Mary:

Don: *‘I’m happy to take them, and just watch the hard evidence of the blood test every 6 months just to see what’s happening, to that cholesterol, level … I would go very much by, you know, what ... those blood levels are telling me.*’

Mary: *‘Yeah, what your body’s doing.*’

Don: *‘Exactly. Because I understand that … er, there is a lot of chemistry there* [laughs] … *and it’s … what those results actually tell me, that lead me down a particular avenue … of taking medication, or not.*’

In a few extreme instances, the power of numbers as effective triggers to action persisted even though the construct being measured was not explicitly mentioned. In their account of Peter’s hospital admission and treatment, for instance, Wendy and Peter carefully recall numbers, but it is opaque (at least to an outsider) to what ‘the numbers’ that triggered action referred:

Wendy: *‘You had, what was it? It was 0.02.*’

Peter: *‘Should be 0.02.*’

Wendy: *And yours was 8.*’

Peter: *‘8.5.*’

Wendy: *‘And then Dr Jones said, when we get to 54 then we start to be a bit concerned.*’

In summary, quantitative information was widely cited, and recalled as effective in triggering action even where participants drew on limited knowledge of what the numbers were measuring or understanding of how threshold levels were chosen. So it seems unlikely that understanding, knowledge, or recall about quantitative information is a *necessary* condition for making a health-related decision, such as taking statins.

### Knowing but not using quantitative risk information

In the few deviant cases where numerical risk information was mentioned in interviews, there was little evidence that it had any particular salience for decision making. Two excerpts from these atypical cases illustrate this. First, Bill recalls his risk of heart disease, but his subsequent comment suggests that what was significant for his decision was not this quantitative estimate, but the inference that this was ‘normal’, in that he was in the middle of a range and like other people his age:

*‘I think I’m on 11% chance of a heart issue in the next 5 years, or something — is that the one? Is that the statistic which is about in the middle isn’t it, I think, for men of my age? So — I think that’s all right.*’

(Bill)

Second, Barbara also described her understanding of numerical risk estimates for heart disease as recalled from her GP’s account. However, the hesitancy of both her recollection and decision suggest that these probabilistic figures were not a major influence on her decision to take statins. Again, her interview suggested that this did not reflect a lack of responsiveness to quantification in general, in that cholesterol readings, in contrast, were reported as being a deciding factor:

*‘He said perhaps I ought to think about going on statins, and he showed me a display on his computer screen, of a hundred hearts, you know showing up percentages and telling me that if I took them for 10 years I would reduce my risk by 4%, from 18 to 14%, or something like that … I think those were the figures. So … er … I wasn’t quite sure whether I wanted to — it didn’t seem a huge … er, difference to me, really … the 4%.*’

(Barbara)

But later:

*‘People talk about different numbers, er, and you don’t really know what they mean, but, my cholesterol had gone up to 9 or something … so I thought perhaps I ought to do something about it.*’

(Barbara)

### Talking about risk and prevention

The rarity with which quantitative risk information appeared in the data cannot be explained by a lack of talk about or interest in reasons to take preventive medication. Indeed, such talk was very prevalent, prompted by the topic guide, but also raised spontaneously:

*Prevention is the ideal, and everything else is, kind of backing up when things go wrong.*’

(Eric)

*‘I think they just said … something to take now, to help you for the future.*’

(Simon)

Explanations like Simon’s, resting simply on being advised that action now would lead to benefit in future, were common. However, the *probability* of experiencing this benefit was rarely mentioned at all in the interview data, and never reported numerically. If future risks were mentioned, it was in generic, non-numeric, terms:

*‘The doctor said to* [my husband], *you know, with blood pressure like this, untreated, you would have a major stroke or heart attack … which could well be fatal.*’

(Hazel)

Further, when asked explicitly, patients rejected the idea that probabilistic quantification would be a useful aid:

Don: *‘It would have to be a very … personal thing, and a GP would have to say ‘Yes! aspirins would definitely help … your particular case.*’

Interviewer: *‘Right. So ‘Out of 100 people exactly like you they’ll help 10’ wouldn’t do it?*’

Don: *‘No. Exactly, that’s right.*’

Two elements appear important here: a personalised message and certainty about the future. Messages recalled as being personal and deterministic were reported as persuasive, as in Larry’s account:

*‘He pointed his finger at me and he said ‘If you want to live a normal life you take the tablets and you’ll live to be an old man … Don’t take the tablets and who knows what will happen.’ So, I have always taken my tablets.*’

(Larry)

Several other interviewees spoke of these elements as essential if advice is to trigger action:

*I don’t particularly like … being put on a regimen of drugs which has been designed for an average person, or a person who falls into a very, very, very large category.*’

(Geoff)

*‘I think we would all be happy to take some things … if we were absolutely convinced that it was necessary, but if it is just a possible thing then you have to think carefully about it.*’

(Liz)

Quantitative risk information was not, it seems, relevant to these two significant components of persuasive knowledge. It was, by definition, related to the population as a whole, not the individual, and it could not be deterministic.

## DISCUSSION

### Summary

Quantitative information about risk was not used in participants’ accounts of how they made the decision about statins. Our analysis suggests that this lack of salience does not reflect lack of knowledge, or aversion to quantitative data in general, as other numbers (such as cholesterol levels) were widely cited as decisive triggers for action. What did trigger decisions to take risk-reducing medication was unequivocal, personalised advice from a doctor.

### Strengths and limitations

Key strengths of this study were that it included both those who did and those who did not decide to take statins, and that it drew on patients’ accounts generated in the settings in which decisions about medication use are likely to be made: the home and between partners. Couple interviews in particular captured the ‘everyday’ talk that underpins people’s day-to-day decision making. The study was limited to residents in East Anglia, a region with relatively low heterogeneity with regard to ethnicity and socioeconomic status; the generalisability of the conclusions to other contexts remains to be tested. That the interviews were conducted by a GP had potential disadvantages in that patients may have been either more reluctant or more anxious to demonstrate detailed understanding of ‘medical’ matters. This was addressed in the analysis by careful consideration of how the interviewer’s expertise was attended to in responses, and by comparing the findings with those from other studies.

### Comparison with existing literature

The contrast between the widespread citation of test results as important for decision making and the almost nonexistent use of quantitative risk information in patients’ accounts has not previously been highlighted in the literature. The scarcity of risk estimates in the data is surprising from a perspective grounded in the risk communication literature. However, this finding is perhaps less surprising in the light of a large body of other research which suggests that people’s health decisions in general rely less on probabilistic future risk estimates, and more on advice that is regarded as applying to them personally,^12^–^17^ and given to them personally by a doctor.^8^,^18^–^22^ Indeed, Gale *et al* found that even people who had been taught how to interpret the results of risk calculations, and who demonstrably understood them, did not base decisions on them, preferring to ask their doctor what to do.^20^ This poses a challenge to the assumption implicit in much biomedical writing about risk communication^2^ that people will heed and use numerical information once they understand it.

That participants do not mention risk estimates when talking about preventive medication, or do not use them as rationales for action, does not of course necessarily mean that these are not part of the useful backdrop of knowledge that patients draw on in making complex decisions about medication use. It is perhaps rare that healthcare decisions are made by patients acting simply as the ‘rational choice agents’ addressed by decision aids and described in game theory,^23^ and in practice a rather broader array of knowledge sources and values are brought into any particular decision. Davison *et al*’s classic work on risk, for instance, identified a ‘lay epidemiology’ in which knowledge about coronary candidacy combined theoretical with experiential knowledge of known others who, for instance, lived to be 100 despite heavy smoking.^24^ Thus there was widespread implicit knowledge of the prevention paradox that interventions which benefit populations may harm individuals, and of the ‘irreducible uncertainty’ of an individual’s future, however definitely statisticians predict outcomes for a large population.^25^

Others^26^–^28^ have since examined the way that people use their practical knowledge alongside theoretical knowledge^29^ from a variety of sources, to produce ‘rules of thumb’ that guide their health decisions; for example, about food choices or taking aspirin. In the process of combining different kinds of knowledge to produce these rules, theoretical knowledge about risk levels is undermined by tacit practical knowledge that the future is uncertain. As Rapley suggests, decisions in practice are ‘distributed’, in that they are ongoing events involving multiple encounters and places.^30^

This study’s data suggest that test results are widely heeded: they are facts about the present, and can be incorporated into an assessment of one’s state of health alongside facts like looking pale or feeling pain.^31^,^32^ It seems plausible that the salience of risk estimates is limited because they are facts about the future, inherently impersonal and uncertain. In building the body of practical knowledge that informs everyday decisions,^33^ such as taking long-term medication, facts about the future may not get a strong foothold, however well they are understood.

### Implications for research and practice

Significant developments in research on cognitive understanding of risk communication have not been matched by our understanding of how such knowledge is used in practice by patients in making decisions about the use of medications such as statins. The finding that people seldom use quantitative risk information in making decisions about preventive interventions, if replicated elsewhere, raises significant questions for both research and practice. Should communicating quantitative information remain a central component of clinicians’ endeavours to facilitate shared decision making?^34^ And should researchers working to make this communication more effective look at outcomes beyond ‘knowledge and understanding’?

If a painstaking discussion of numerical risk information has little or no effect on the patient’s decision, a cash-limited health service may decide to look for shortcuts to a patient-centred approach.^35^ One such shortcut might be found by following Elwyn *et al*’s recommendation to offer a choice about participation in decision making.^36^ For instance, saying ‘I would suggest you give statins a try, so as to make you less likely to have heart attacks in future — would you like me to show you the statistics about that?’ may often elicit the reply ‘No thanks’, shortening the consultation without making any difference to its outcomes.

Such an approach is arguably more patient-centred than attempts to ‘engage … patients in risk management discussions’,^5^ because it recognises a potential misunderstanding that may undermine the project of risk communication in the context of prevention. To a clinician, preventive medication is synonymous with risk-reducing medication, but this small study suggests that patients may not consider ‘prevention’ in quite the same way. At least in the context of preventive medication, some people may not base decisions on the probabilities of various possible outcomes, however effectively those probabilities are explained or carefully weighted to reflect individual preferences and values.

In summary, recommended approaches to communicating about risk are challenged by this study’s finding that patients make minimal use of quantitative risk estimates when talking about preventive medication.
